# Potential role of MELD and MAP18 in patients with structural temporal lobe epilepsy

**DOI:** 10.1007/s00234-025-03549-6

**Published:** 2025-03-05

**Authors:** Francesca De Luca, Jose Carlos Pariente, Sofia González-Ortiz, Estefanía Conde-Blanco, Mar Carreño, Xavier Setoain, Nuria Bargalló

**Affiliations:** 1 Department of Neuroradiology, Karolinska University Hospital, Stockholm, Sweden; 2 Department of Clinical Neuroscience, Karolinska Institute, Stockholm, Sweden; 3 Fundació de Recerca Clínic Barcelona, Institut d’Investigacions Biomèdiques August Pi i Sunyer, Barcelona, Spain; 4 Department of Neuroradiology, Diagnostic Imaging Centre, Hospital Clinic, Barcelona, Spain; 5 Department of Neurology, Hospital Clinic, Barcelona, Spain; 6 European Reference Network EpiCARE, Barcelona, Spain; 7 Nuclear Medicine Department, Diagnostic Imaging Centre, Hospital Clinic, Barcelona, Spain; 8 Biomedical Research Networking Center in Bioengineering, Biomaterials and Nanomedicine (CIBER-BBN), Barcelona, Spain; 9 Centro de Investigación Biomédica en Red de Salud Mental (CIBERSAM), Madrid, Spain

**Keywords:** MAP18 Morphometric analysis program, MELD Multi-centre epilepsy lesion detection, TLE Temporal lobe epilepsy

## Abstract

**Purpose:**

This study compared two image post-processing toolboxes primarily designed for focal cortical dysplasia (FCD): Multi-Centre Epilepsy Lesion Detection (MELD) and Morphometric Analysis Program (MAP18), in identifying temporal lobe epilepsy (TLE) structural lesions on MRI.

**Methods:**

This retrospective study examined 79 adults, 58 patients with confirmed TLE, and 21 healthy controls. All participants underwent an elective brain MRI between June 2007 – May 2023 at Hospital Clinic, Barcelona, Spain. All the 3D T1-weighted images were processed using MELD and MAP18 to detect potential epileptogenic lesions. The location (lateral or mesial) and laterality of the reference TLE structural lesion (refTLE) were determined through histopathology or multidisciplinary consensus based on clinical data. Toolboxes’ performance was evaluated using descriptive statistics, specificity, and diagnostic accuracy. Additionally, a second-look MRI was conducted for cases where abnormalities detected by MELD and MAP18 did not match the refTLE.

**Results:**

MELD and MAP18 demonstrated variability in specificity and diagnostic accuracy. Specificity ranged from 48% to 86%, with ProbMAP (MAP18) achieving the highest values. Global diagnostic accuracy ranged from 7% to 42%, with MELD showing the highest performance. In four patients with visible epileptogenic lesions on MRI, MELD and MAP18 identified additional abnormalities that were previously overlooked. Moreover, MELD detected one TLE lesion in one patient initially classified as MRI–negative (nonlesional).

**Conclusion:**

Incorporating tools like MELD and MAP18 into the diagnostic workflow can enhance the detection of TLE-related abnormalities on MRI, potentially improving patient outcomes and aiding in clinical decision-making.

**Supplementary Information:**

The online version contains supplementary material available at 10.1007/s00234-025-03549-6.

## Introduction

Temporal lobe epilepsy (TLE) is a prevalent form of epilepsy, accounting for approximately 30% of adult epilepsy [1, 2], characterized by a complex etiology involving different structural lesions within the temporal lobe at either mesial temporal lobe (MTL), lateral temporal lobe (LTL), or concomitant lesions at MTL and LTL [3], which can be confirmed by histopathology. Recent guidelines [4] recommended using structural MRI to detect TLE structural lesions routinely on imaging. Evidence suggests that most TLE structural lesions confirmed in histopathology have corresponding substrates on imaging [5]. However, TLE structural lesions can be subtle or not visible [6, 7] on MRI, which might lead to overlooking these findings, even by experienced readers.

Hippocampal sclerosis (HS) is a predominant lesion associated with temporal lobe epilepsy (TLE), characterized by neuronal loss, gliosis, and hippocampal atrophy. It may occur in isolation or in conjunction with other abnormalities such as focal cortical dysplasia (FCD) [8], or grey-white matter blurring (GMB), and atrophy of the temporal pole [9] While HS is often evident on MRI, FCD, particularly FCD Type 1 and GMB, frequently eludes detection on MRI due to their subtle presentation. Another potential epileptogenic lesion that can be overlooked in TLE is a small sphenoid encephalocele, allowing a small part of the temporal lobe to herniate through a defect or abnormal opening in the base of the skull at the sphenoid bone [10] Other lesions linked to TLE, such as low-grade epilepsy-associated tumors (LEATs), including gangliogliomas, dysembryoplastic neuroepithelial tumors (DNETs), and pleomorphic xanthoastrocytomas, generally exhibit distinct MRI features, facilitating differentiation from other TLE-related lesions [11].

TLE structural lesions often exhibit drug resistance, underlining the importance of surgical evaluation [12, 13]. Accurate localization of TLE structural lesions on imaging is crucial for planning surgical treatment and can significantly affect the treatment outcome. It has been shown that the detection of TLE structural lesions on MRI and complete surgical resection increases the likelihood of patients achieving seizure freedom [14–16]. Conversely, negative MRI [7] and incomplete or non-resection of TLE structural lesions are associated with a less favorable treatment outcome [17]. Negative MRI – namely, the absence of detectable structural lesions on MRI in the presence of TLE epilepsy – yet represents a challenge in epilepsy imaging and highlights the need to incorporate more advanced imaging algorithms for a higher detection of epileptogenic foci on MRI [18, 19].

Several algorithms for image post-processing have been investigated in the literature to enhance radiologists' ability to detect epilepsy-related lesions on MRI [20], primarily to enhance the detection of focal cortical dysplasia (FCD) in neocortical epilepsy. Among these, two image post-processing algorithms, Multi-Centre Epilepsy Lesion Detection (MELD) [21] and Morphometric Analysis Program (MAP18) [22], have demonstrated the ability to increase the diagnostic yields of MRI epilepsy-related lesions. Both toolboxes were specially designed for the detection of FCD, MELD utilizing data based on individual vertices in a mesh representation of the brain (vertex-wise analysis), whereas MAP18 uses data within the brain volumetric space (voxel-based analysis).

None of these two tools, trained to detect focal cortical dysplasia (FCD), has been evaluated for the detection of other structural lesions beyond FCD and associated with TLE. The detection of these other abnormalities, such as FCD type I and GBM, is important given their often inconspicuous appearance on MRI. Thus, validating these tools for the detection of a broader spectrum of TLE structural lesions could enhance diagnostic accuracy and clinical management in TLE.

This retrospective study compared the accuracy of MELD and MAP18 in detecting MRI lesions in adult patients with TLE.

## Materials and methods

This retrospective study was designed and reported according to the up-to-date diagnostic accuracy STARD guidelines [23].

### Cohort

This retrospective study analyzed data from 79 adults who underwent elective brain MRI at the epilepsy unit of Hospital Clinic, Barcelona, Spain, between June 2007 and May 2023. The cohort included 58 patients with confirmed TLE and 21 healthy controls with no neurological or psychiatric disorders (HC). Inclusion criteria for the study required all participants to be ≥18 years of age at the MRI examination and availability of an MRI exam that included a 3D T1-weighted image. Exclusion criteria were diagnosis of epilepsy other than TLE and unavailability of 3D T1-weighted image. Relevant clinical and radiological information was retrieved from the patient's medical records. The study was approved by the Hospital Clinic Barcelona Ethics Committee (HCB 2022/1225) and complied with the Declaration of Helsinki. Informed consent was waived, given the retrospective nature of the study.

### MRI acquisition

All MRI scans were performed on either a 3T Magnetom Tim Trio or a Prisma Fit scanner from Siemens, located at the Diagnostic Imaging Center of Hospital Clinic, Barcelona, Spain. All included subjects underwent a 3D structural, high-resolution T1-weighted scan according to the diagnostic epilepsy protocol for clinical purposes. Supplementary Table S1 outlines detailed information about MRI acquisition parameters.

### MELD and MAP18 post-processing

All T1-weighted images were processed using two post-processing analysis tools, MELD (version 1.1.0) and MAP18 (compiled version as of 2023/04/23). Before initiating the image post-processing, all T1-weighted images were evaluated for image quality to ensure the reliability of the subsequent analyses. MAPcombined refers to a map generated by MAP18, which selects, for each voxel, the highest z-score among three feature parameter maps – namely “junction" (blurring at the grey-white matter junction), "extension" (abnormal extensions of grey matter into white matter), and "thickness" (variations in cortical thickness). A threshold z-score of 4 [24], provided as an output image directly from MAP18, was used to distinguish those values significantly deviating from the normative database of controls (NDBs), which consisted of subjects from the Diagnostic Imaging Center of the Hospital Clinic, Barcelona, Spain. This process generates a single combined map for each subject, highlighting the most significant abnormalities across these parameters (junction, extension, thickness) that may be associated with epileptic lesions. Two scanner-specific NDBs were used: one for controls acquired using the Prisma scanner and another for controls acquired using the Trio scanner. However, for one specific 3D T1-weighted sequence used on the Trio scanner, no normative controls were available in the database. While this limitation was noted, all available data were processed using the corresponding scanner-specific NDB to minimize false-positive findings related to scanner-specific variability.

In contrast, both ProbMAP and MELD employ Artificial Neural Network (ANN) to detect possible epileptic foci. While ProbMAP utilizes the previously mentioned feature parameters to train the ANN [25], MELD incorporates a distinct set of surface-based features obtained from the cortical reconstruction from FreeSurfer processing. In this study, the chunked version of the ProbMAP ANN, implemented in the 2023 compilation, was used. For MELD, the meld classifier ANN from version 1.1.0 was employed. Detailed information about the post-processing pipelines can be found in [21] and [22]. Subjects included in the cohort of this study were not used to establish the NDBs nor to train the ANN models for both MELD and MAP18 methodologies.

### Reference standard for TLE Lesion (refTLE)

In this study, similarly to a previous study evaluating the performance of MAP [26], the reference standard for the location and laterality of the TLE structural lesion (refTLE) was established through two methods:

- for patients who underwent surgical intervention, histopathology served as the basis for refTLE.
- for patients where surgery was not performed, refTLE was established through a multidisciplinary consensus, incorporating a detailed clinical workup, including a range of diagnostic tests such as video-EEG, EEG, PET, SPECT/SISCOM, and neuropsychological tests.

Structural lesion laterality was defined as unilateral (left or right) or bilateral. Once the location and laterality were defined, the TLE structural lesions were classified based on four distinct categories as follows: 1) MTLE (located in the hippocampus, parahippocampus, amygdala, and entorhinal cortex; 2) LTLE (located in the superior-, inferior-, middle temporal gyrus, fusiform gyrus, temporal operculum, and insula; 3) concomitant MTLE/LTLE, indicating the co-presence of MTLE and LTLE, and 4) indeterminate temporal (iTLE), when the localization is confirmed to be in the temporal lobe but could not be precisely localized to a specific area.

### MELD and MAP18 image analysis

An experienced radiology reader with five years of radiology experience analyzed the post-processed maps. Abnormalities detected by MELD were reported according to the software-generated post-processing report, which provides anatomical localization and laterality. For MAP18, a visual assessment of the abnormalities was performed on the so-called “FCD probability” (called in this study “ProbMAP”) [25] and "combined z-score 4" (called in this study “MAPcombined”) [24] maps of the toolbox. This assessment was performed on ITKSNAP (version 3.6.0 (http://www.itksnap.org). Abnormalities detected by MELD and MAP18 were classified using the same categories established for refTLE. When an abnormality was detected outside the TL, it was classified according to the brain anatomy as frontal, parietal, occipital, cingulate, basal ganglia, corpus callosum, and periventricular white matter.

### Statistical analysis

To evaluate the performance of MELD and MAP18 in identifying possible TLE structural lesions, the anatomical localization and laterality of the abnormalities observed by MELD and MAP18 were compared to the refTLE. This comparison aimed to determine the specificity and diagnostic accuracy of these tools. Results were summarized using contingency tables, and statistical analysis was conducted with an in-house Python script (version 3.7, http://www.python.org).

### Specificity and diagnostic accuracy

Similar to a previous study [27], specificity was determined by analyzing control cases that were expected to have no findings. For this purpose, abnormalities detected by MELD and MAP18 in the healthy control cases were categorized as false positives (FP). The specificity was then calculated using the formula: Specificity = TN / TN + FP, where TN represents the true negatives, or control cases without abnormalities detected, and FP represents the false positives.

To assess diagnostic accuracy, the localization and laterality of the abnormalities identified by MELD and MAP18 were compared against the refTLE classifications (MTLE, LTLE, MTLE/LTLE, iTLE). The findings were evaluated as either concordant or discordant with the refTLE [27] according to two distinct conditions to refine the evaluation of lesion detection efficacy:

- Strict agreement, MELD and/or MAP18 precisely detected abnormalities as per refTLE, without additional false positive lesions.
- Permissive agreement, MELD and/or MAP18 correctly detected the lesion corresponding to the refTLE but also detected other, non-corresponding false positive lesions.

The diagnostic accuracy was calculated using the formula: Diagnostic accuracy = TP + TN / total cases per evaluated group, where TP represents the true positives, and TN represents the true negatives.

### Second look image analysis

For patients where MELD and MAP18 detected FP abnormalities at the TL but outside the specified refTLE regions, an experienced neuroradiologist with 32 years of experience in MRI imaging performed a second-look MRI. This aimed to uncover any TLE structural lesions that may have been missed earlier in the clinical workup and could be detected retrospectively with the guidance of MELD and MAP18 findings.

## Results

### Cohort characteristics and refTLE

Detailed information about the cohort’s characteristics, including the refTLE, can be found in Table 1. Among the cohort, bilateral pathology was found in three cases: one with MTLE (bilateral MTS), one with MTLE/LTLE (bilateral MTS and unilateral cortico-subcortical gliosis at the right side), and one with TLE (Video EEG findings suggestive of bilateral TLE, although negative MRI). Another intriguing case involves a patient with MTLE/LTLE (low-grade glioma), for whom two MRI scans obtained years apart (2011 and 2019) were analyzed with post-processing. These scans were included since the radiological features of the tumor were different between the scans (subtle on MRI 2011, clearly visible on MRI 2019). All TLE structural lesions that underwent a neuropathological examination were classified according to the neuropathological classification system at the year of diagnosis.

**Table 1:** Cohort's characteristics

**Subjects, n 79**	
Age years mean (SD, range)	37 (11, 19–64)
Sex female, n (%)	47 (60)
Controls, n (%)	21 (27)
Patients, n (%)	58 (73)
**MRI scans, n 80**	
**MRI scans of patients, n 59**	
refTLE^a^ category, n MRI scans (%)	
MTLE	13 (22)
MTS	12
DNET/glioma on MRI	1
LTLE	9 (15)
LTL cortico-subcortical gliosis	3
LTL encephalocele	2
Low-grade neuroepithelial tumor	1
LTL cortical dysplasia	1
Cavernoma	1
Negative MRI, no surgery^b^	1
MTLE/LTLE	33 (56)
MTS with concomitant ipsilateral LTL gliosis^c^	25
MTS and ipsilateral LTL cortex dysplasia^d^	2
Hamartoma at the hippocampal head and ipsilateral LTL cortical dysplasia	1
Dysplasia of the amygdala and ipsilateral LTL cortical dysplasia^e^	1
Ipsilateral DNET at the MTL, MTS and LTL gliosis	1
Grade 2 glioma^f^	2
Limbic encephalitis	1
iTLE	4 (7)
Anatomical variants at the MTLE on MRI^gh^	1
Negative MRI^hi^	3
Surgery, n patients (%)	30 (58)
with negative MRI	3^cde^
Negative MRI, n patients (%)	7 (12)
LTLE^b^	1
MTLE/LTLE^cde^	3
iTLE^f^	3

*MTLE* mesial temporal lobe epilepsy; *MTL* mesial temporal lobe; *MTS* mesial temporal sclerosis; *LTLE* lateral temporal lobe epilepsy; *LTL* lateral temporal lobe; *MTLE/LTLE* concomitant mesial and temporal lobe epilepsy; SD= standard deviation; TL= temporal lobe; *iTLE* indeterminate temporal lobe epilepsy where the multidisciplinary consensus could not further characterize the lesion type within the temporal lobe

^a^According to the information from the multidisciplinary consensus in the clinical charts

^b^Video EEG suggestive of LTLE

^c^One of the 23 cases had a negative MRI and underwent surgery

^d^One of the two cases had a negative MRI and underwent surgery

^e^This case had a negative MRI and underwent surgery

^f^Same patient who underwent two different MRI examinations years apart (2011 and 2019)

^g^MRI suggestive of concomitant left hippocampal malrotation and right amygdala hypertrophy, although no radiological findings of MTS

^h^All cases had a positive video EEG or EEG

^I^Two cases had a positive SPECT/SISCOM

### Control group analysis: false positive rate and specificity

Among the group of control cases, FP rates varied significantly across the toolboxes: MAPcombined reported the highest at 52%, followed by MELD with 43%, and ProbMAP with 29%. The specificity obtained on the toolboxes was 71% for ProbMAP, 57% for MELD, and 48% for MAPcombined. When merging the post-processing information from multiple maps, the specificity rate increased by 76% for MAP18 (ProbMAP + MAPcombined) and 86% for MELD + MAP18. Within these FP abnormalities, MAPcombined identified 22 bilateral lesions, ProbMAP identified 5, and MELD did not identify any bilateral lesions. Thirty-three percent of the control cases were accurately identified without any abnormality by all three diagnostic tools. The exact location of the FP abnormalities in the control group is shown in detail in Table 2.

**Table 2:** FP abnormalities detected by MELD, ProbMAP, and MAPcombined in the control group

FP lesions in controls, n	MELD	ProbMAP	MAPcombined
Frontal lobe	2	2	8
Parietal lobe	4	5	7
Temporal lobe	4	3	7
Occipital lobe	0	2	5
Cingulate	2	0	2
Corpus callosum	0	0	11
Periventricular white matter	0	0	6
Basal ganglia	0	0	6

### Patients group analysis: diagnostic accuracy at detecting TLE

**Fig. 1:**
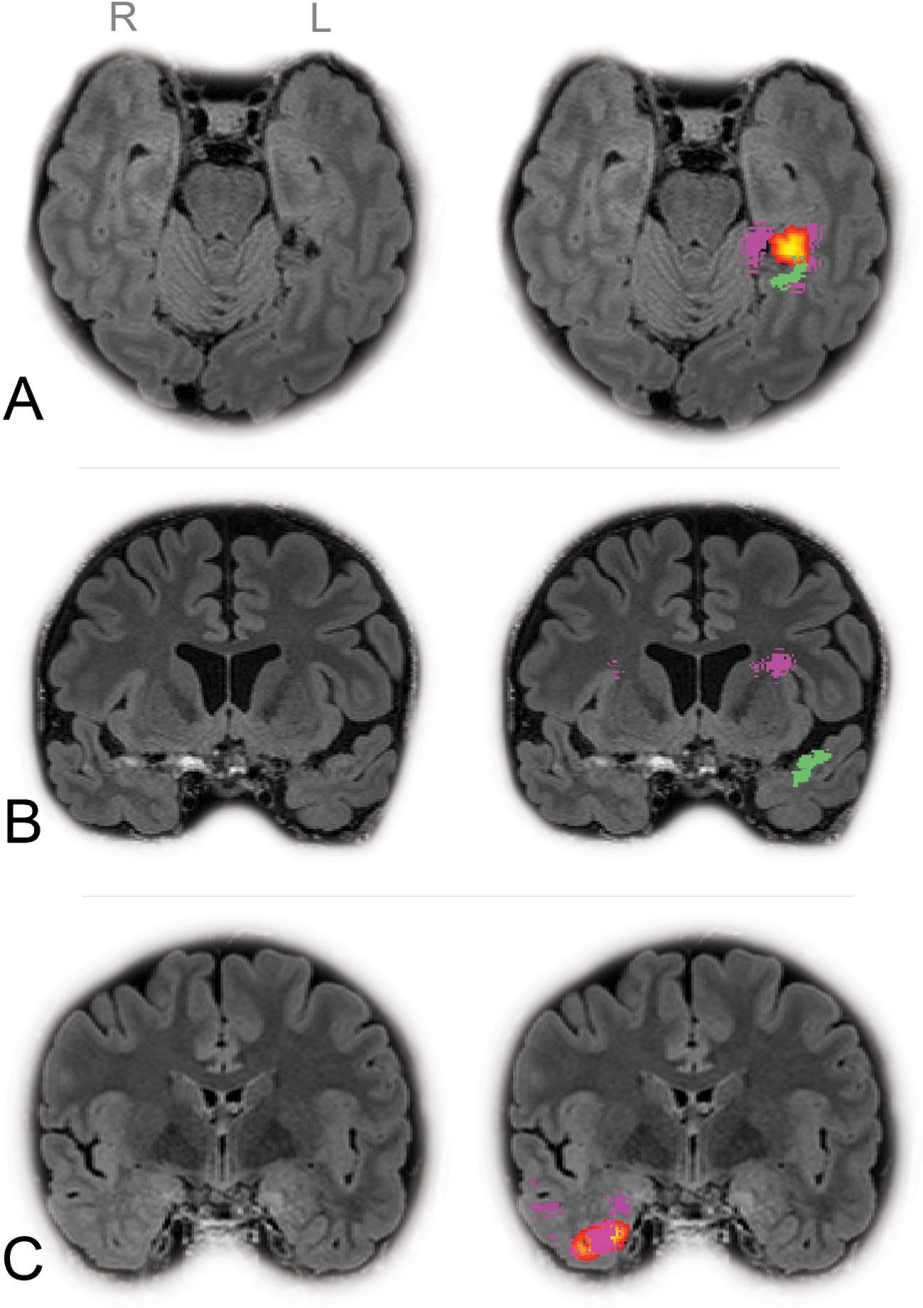
Examples of different TLE lesions identified on patients, with FLAIR images on the left side and their overlaid abnormalities detected by the toolboxes on the right side: MELD (green), ProbMAP (orange), and MAPcombined (purple). (A) A case of low-grade neuroepithelial tumor located at the posterior basal aspect of the left temporal lobe, classified as refTLE “LTLE”. A correct localization of the lesion responsible for the TLE was found in all three maps: MELD, ProbMAP, and MAPcombined. (B) A case of left MTS with gliosis. MELD detected abnormality at the left parahippocampus (cluster not shown in the image) as well as the left temporal pole (cluster shown in the image), while ProbMAP didn’t show any abnormality and MAPcombined demonstrates FP clusters bilaterally outside the temporal regions. (C) A case of right MTS and gliosis of the anterior temporal pole confirmed by histology. The lesion is correctly detected by MAP18 (ProbMAP and MAPcombined). MELD identified an FP lesion at the caudal anterior cingulate SIN (cluster not shown)

The diagnostic accuracy for patients with TLE across the four refTLE categories varied significantly between the toolboxes. Under the strict agreement condition, accuracy rates reached up to 10% for MELD and 7% for ProbMAP and MAPcombined. These rates increased under the permissive agreement condition up to 15% for MELD, 12% ProbMAP, and 31% MAPcombined, as detailed in Table 3. Adopting a more flexible approach and merging post-processed maps significantly improved the overall diagnostic accuracy, up to 42% when combining the three post-processing maps (MELD, ProbMAP and MAPcombined), and 34% when using both MAP18 maps, as shown in Supplementary Tables 2-3. LTLE had the highest diagnostic accuracy rates among the refTLE categories, reaching up to 44% with MELD under a strict agreement condition and up to 67% at a permissive agreement condition. Examples of correctly identified TLE structural lesions across the maps are illustrated in Figure 1. Detailed results for FP (strict agreement condition) and FN cases in the group of patients with confirmed TLE are provided in Supplementary Table 4 and Supplementary Figure 1. Detailed results for FN cases are provided in Table 3.

**Table 3:**
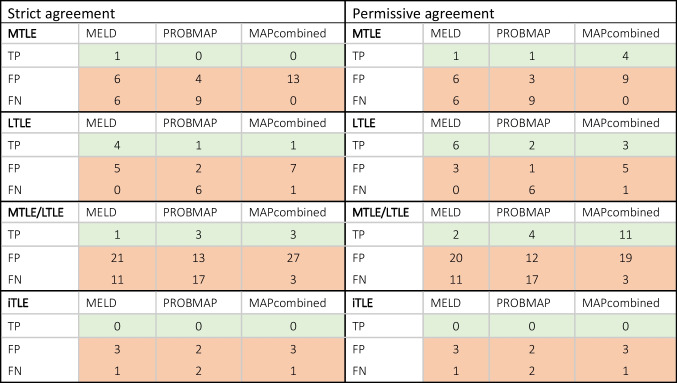
Pivot table for TP, FP, and FN in MELD, ProbMAP, and MAPcombined in the different refTLE categories and agreement conditions

### Second look MRI

**Fig. 2:**
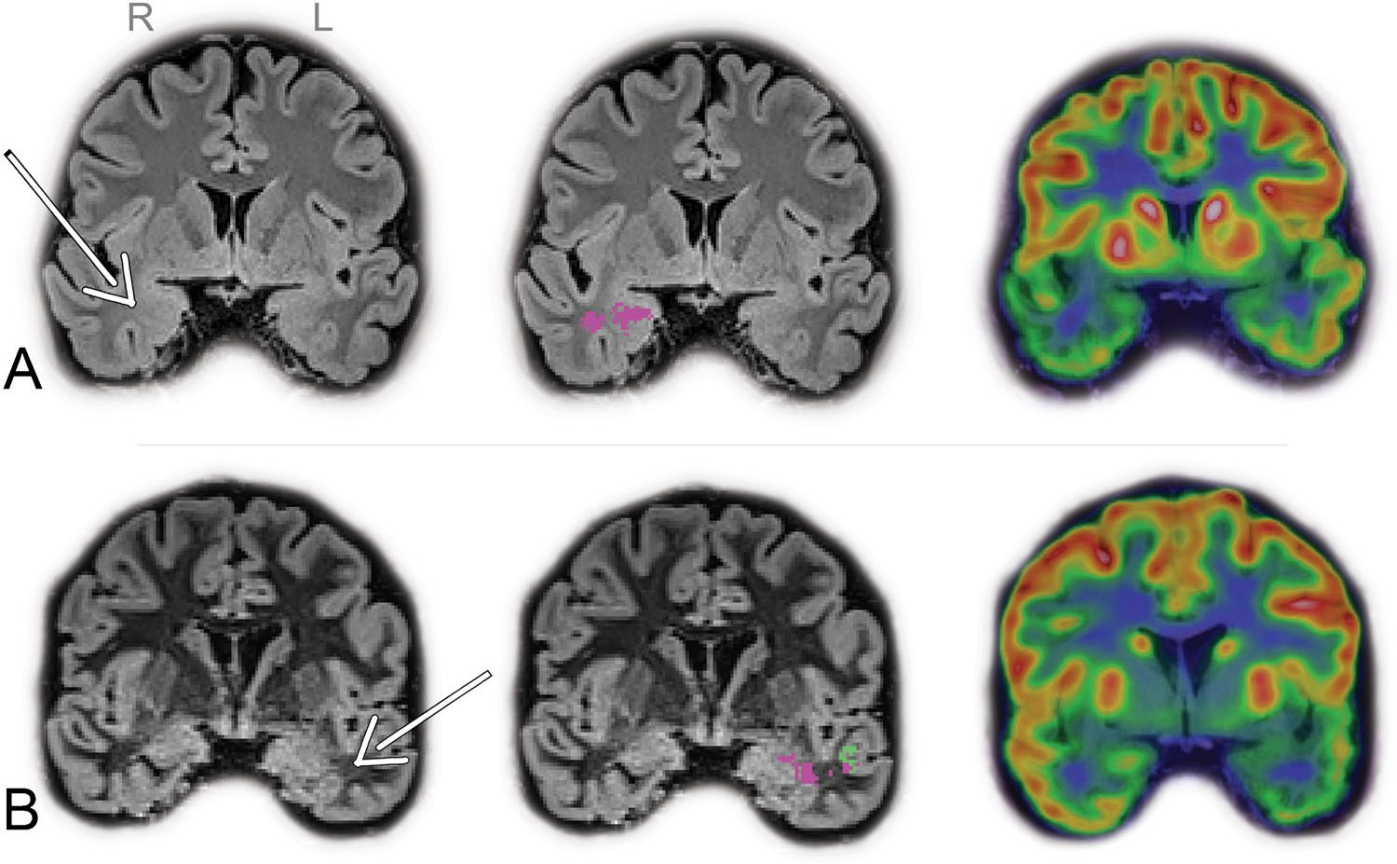
Examples of additional lesions identified on second look MRI, showcasing FLAIR/DIR images on the left side, FDG-PET on the right side, and their overlaid abnormalities detected by the toolboxes in the middle: MELD (green), and MAPcombined (purple). (A) A patient with right TLE in which only right hippocampal sclerosis was initially detected during the clinical work-up. MELD and MAPcombined revealed abnormal clusters in the right temporal pole. (MELD cluster located more anteriorly to the coronal slice not shown). The second look MRI confirmed the presence of a subtle blurring between the grey and white matter in the right temporal pole (arrow), a finding concordant with the hypometabolism observed in FDG-PET imaging in the same area of the right temporal pole. (B) A patient with left TLE where initial MRI findings were limited to left hippocampal sclerosis. Abnormal clusters were identified in the left amygdala (MAPcombined) and white matter of the left temporal pole (MELD and MAPcombined). The second look on MRI confirmed the presence of a subtle increased signal intensity in the white matter of the left temporal pole (arrow), consistent with the hypometabolism seen in the corresponding area in the FDG-PET image

A second look MRI was conducted for patients with FP lesions detected by MELD and MAP18 at the TL, but outside the refTLE. In four patients, abnormalities in TLE identified by MELD and MAP18 were confirmed as true positive upon the second look. Initially, the refTLE was initially defined as MTS-only in two patients (one case on the right side; one case on the left side), as shown in Figure 2. The second look confirmed the presence on MRI of additional subtle TLE abnormalities in the LTL on the same side as the MTS pathology. Notably, MELD failed to detect abnormalities in the mesial region in both cases, which were found by MAPcombined. In another case, the refTLE was originally classified as left LTLE (neocortical epilepsy). MAPcombined correctly identified the refTLE and an additional abnormality on the MTL, which was also confirmed by the second look MRI. Lastly, in the only case with bilateral MTS pathology with unilateral right-sided cortico-subcortical gliosis, MAPcombined detected an additional left-sided cortico-subcortical abnormality, confirmed by the second look. However, MAPcombined correctly identified the MTS pathology only on the right side.

## Discussion

This retrospective study explored the ability of two predictive toolboxes, MELD and MAP18, to detect MRI TLE structural lesions in a mixed cohort of healthy controls and patients with clinically confirmed TLE. To our knowledge, this is the first study comparing the use of MELD and MAP18 in detecting MRI TLE lesions, including MTLE, LTLE, and concomitant MTLE/LTLE lesions.

MELD and MAP18 demonstrated varying specificity and diagnostic accuracy rates across the cohort, with ProbMAP achieving the highest specificity at 71%. In contrast, MAP18 showed the lowest specificity due to a higher rate of false positives, which could complicate image interpretation. However, combining the three approaches (MELD, ProbMAP, and MAP18combined) consistently enhanced the specificity, reaching up to 86%. This combined approach underscores its clinical utility, offering radiologists increased confidence in ruling out epilepsy lesions in patients with suspected epilepsy. The observed specificity rates align with prior studies on MELD [21] and MAP18 [26].

In epilepsy patients in this study, the algorithms demonstrated a global diagnostic accuracy of up to 10% at a strict agreement with refTLE, increasing to 31% at a permissive agreement. These findings vary from previously reported rates [21, 22, 26, 28–31], probably because in our study we included a mixed cohort with heterogeneous TLE structural lesions, encompassing both mesial and lateral pathologies. The MELD [21] approach, which relies on cortical surface reconstruction, posed challenges in identifying lesions located in mesial and subcortical structures, potentially impacting global accuracy. In contrast, MAP18 [22], employing a voxel-based methodology, successfully identified some hippocampal sclerosis lesions, consistently with prior studies [26].

Our study demonstrated that integrating multiple post-processing maps consistently enhanced the diagnostic accuracy compared to using individual maps alone. Notably, the analysis elucidated that the LTLE region emerged as the most accurately diagnosed area, with MELD achieving an accuracy rate of up to 67%, which rose to 77% upon a combination of all three post-processing maps. This notable diagnostic accuracy observed within the LTLE region aligns with findings from previous studies evaluating FCD [26] , a result that was anticipated given the algorithms being specifically trained to identify characteristics typically associated with FCD [21, 22].

A second look MRI identified additional TLE lesions in four patients, previously overlooked by the radiologists during the clinical assessment but detected through MELD and MAP18 post-processing maps. These patients had a positive MRI and clinically confirmed TLE but were classified as nonsurgical candidates in the multidisciplinary team meeting. Notably, detecting these additional TLE lesions by MELD and MAPcombined established a new refTLE category (MTLE/LTLE) for all four patients, potentially influencing their surgical management. Furthermore, in one patient with a negative (nonlesional) MRI, MELD identified an LTLE lesion that corresponded in location and laterality with findings from PET and video-EEG. These findings highlight the potential utility of MELD and MAP18 post-processing for both nonlesional [26, 32, 33] and lesional MRI.

Consistent with a previous study [29], this study included only post-processing maps with pre-established statistical threshold (ANN for ProbMAP and 4*z-score for MAPcombined), enhancing the reproducibility and replicability of our results. Further, applying statistically validated thresholds for post-processing maps contributes to time efficiency by streamlining the clinical workflow, reducing the time required for radiologists to assess the lesions detected by MELD and MAP18 during the diagnostic workup.

In this study, the agreement between MELD and MAP18 post-processing maps was low in identifying the same healthy control cases (true negatives) and the same TLE patient cases (true positives). This result is not unexpected, as the post-processing maps were developed using different training models, resulting in variations in their ability to detect distinct types of TLE lesions.

A limitation of the study is its retrospective design, as subject inclusion depended on the availability of T1-weighted images with sufficient image quality for post-processing. This requirement reduced the sample size, potentially introducing selection bias. Nevertheless, the study encompassed a broad spectrum of TLE pathologies, including MTLE, LTLE, and combined MTLE/LTLE, enhancing the generalizability of our results. Another limitation of the study is the scanner-specific variability in the NDBs used in MAP18. While two separate NDBs were created, one for controls acquired with the Prisma scanner and another for the Trio scanner, there was one specific 3D T1-weighted sequence for which no controls were available in the Trio NDB. This limitation could potentially impact the reliability of the results derived from that sequence. While scanner-specific NDBs do not necessarily increase sensitivity, they help reduce false-positive findings caused by scanner-specific signal inhomogeneities. Future studies incorporating more comprehensive scanner-specific NDBs could further strengthen the robustness of the findings. In summary, the variability in specificity, the retrospective nature of the study, and scanner-specific limitations indicate the need for further research to validate these findings and refine the algorithms for broader clinical applications. Future studies incorporating larger, more diverse cohorts and improved normative databases could enhance the reliability and robustness of these diagnostic tools.

## Conclusions

This study highlighted the potential of integrating post-processing tools, MELD and MAP18, into routine diagnostic workflows for temporal lobe epilepsy (TLE). While specificity and diagnostic accuracy varied across the two algorithms, the combined use of MELD and MAP18 enhanced detection capabilities, particularly in subtle or nonlesional TLE cases, offering valuable insights for clinical decision-making.

## Supplementary Information

Below is the link to the electronic supplementary material.

Supplementary file1 (DOCX 185 KB)

## Data Availability

All data used and/or analyzed during the current study are available from the corresponding author, Francesca De Luca, upon reasonable request.

## Abbreviations

MAP18: Morphometric analysis program
MELD: Multi-centre epilepsy lesion detection
TL: Temporal lobe
TLE: Temporal lobe epilepsy

## Declarations

### Scientific presentation

Part of this study's results were presented as a scientific poster at the Annual Meeting of the European Society of Neuroradiology, held in Wien in September 2023, and at the Annual Meeting of the Spanish Society of Neuroradiology, held in October 2023.
