# Supplementary material for: Potential role of MELD and MAP18 in patients with structural temporal lobe epilepsy
Source: Neuroradiology. 2025 Mar 5;67(4):865–74. doi: 10.1007/s00234-025-03549-6 (PMC12041102; doi:10.1007/s00234-025-03549-6)

Table S1. MRI acquisition protocols

|  | **Protocol #1** | **Protocol #2** | **Protocol #3** | **Protocol #4** | **Protocol #5** |
| --- | --- | --- | --- | --- | --- |
| Scanner | TrioTim (Siemens) | TrioTim (Siemens) | TrioTim (Siemens) | TrioTim (Siemens) | PrismaFit (Siemens) |
| Magnetic field | 3T | 3T | 3T | 3T | 3T |
| Orientation | Coronal | Coronal | Coronal | Sagittal | Sagittal |
| Vx size (mm^3^) | 0.94x0.94x, 0.90 | 0.9x0.9x1.2 | 0.86x0.86x0.9 | 1x1x1 | 0.8x0.8x0.8 |
| FOV (mm^2^) | 240x210 | 230x200 | 220x192 | 256x256 | 256x240 |
| Acquisition matrix | 256x224 | 256x224 | 256x224 | 256x256 | 320x300 |
| Slices | 240 | 192 | 240 | 208 | 208 |
| TE (ms) | 3.01 | 3.01 | 3.01 | 2.98 | 2.22 |
| TR (ms) | 2000 | 2000 | 2000 | 2300 | 2400 |
| TI (ms) | 900 | 900 | 900 | 900 | 1000 |
| FA (º) | 9 | 9 | 9 | 9 | 8 |

Table S2. TP cases when combining MELD and MAP18 (ProbMAP and MAP combined) post-processing.

| refTLE category | TP (n), strong agreement | TP (n), slight agreement |
| --- | --- | --- |
| MTLE, n 13 | 1 | 5 |
| LTLE, n 9 | 4 | 7 |
| MTLE/LTLE, n 33 | 5 | 13 |
| TLE, n 4 | 0 | 0 |

Table S3. TP cases when combining MAP18 (ProbMAP and MAP combined) post-processing.

| refTLE category | TP (n), strong agreement | TP (n), slight agreement |
| --- | --- | --- |
| MTLE, n 13 | 0 | 4 |
| LTLE, n 9 | 1 | 4 |
| MTLE/LTLE, n 33 | 4 | 12 |
| TLE, n 4 | 0 | 0 |

Table S4. FP abnormalities (strict agreement condition) detected by MELD, ProbMAP, and MAP combined in the group of patients with confirmed TLE.

| **FP lesions in patients, n** | **MELD** | **ProbMAP** | **MAP combined** |
| --- | --- | --- | --- |
| Frontal lobe | 9 | 11 | 33 |
| Parietal lobe | 11 | 10 | 22 |
| Temporal lobe | 17 | 6 | 26 |
| Occipital lobe | 0 | 5 | 9 |
| Cingulate | 7 | 0 | 3 |
| Corpus callosum | 0 | 0 | 11 |
| Periventricular white matter | 0 | 1 | 23 |
| Basal ganglia | 0 | 0 | 8 |

Figure S1. Graphical representation of FP abnormalities (strict agreement condition) detected by MELD, ProbMAP, and MAP combined in the group of patients with confirmed TLE.


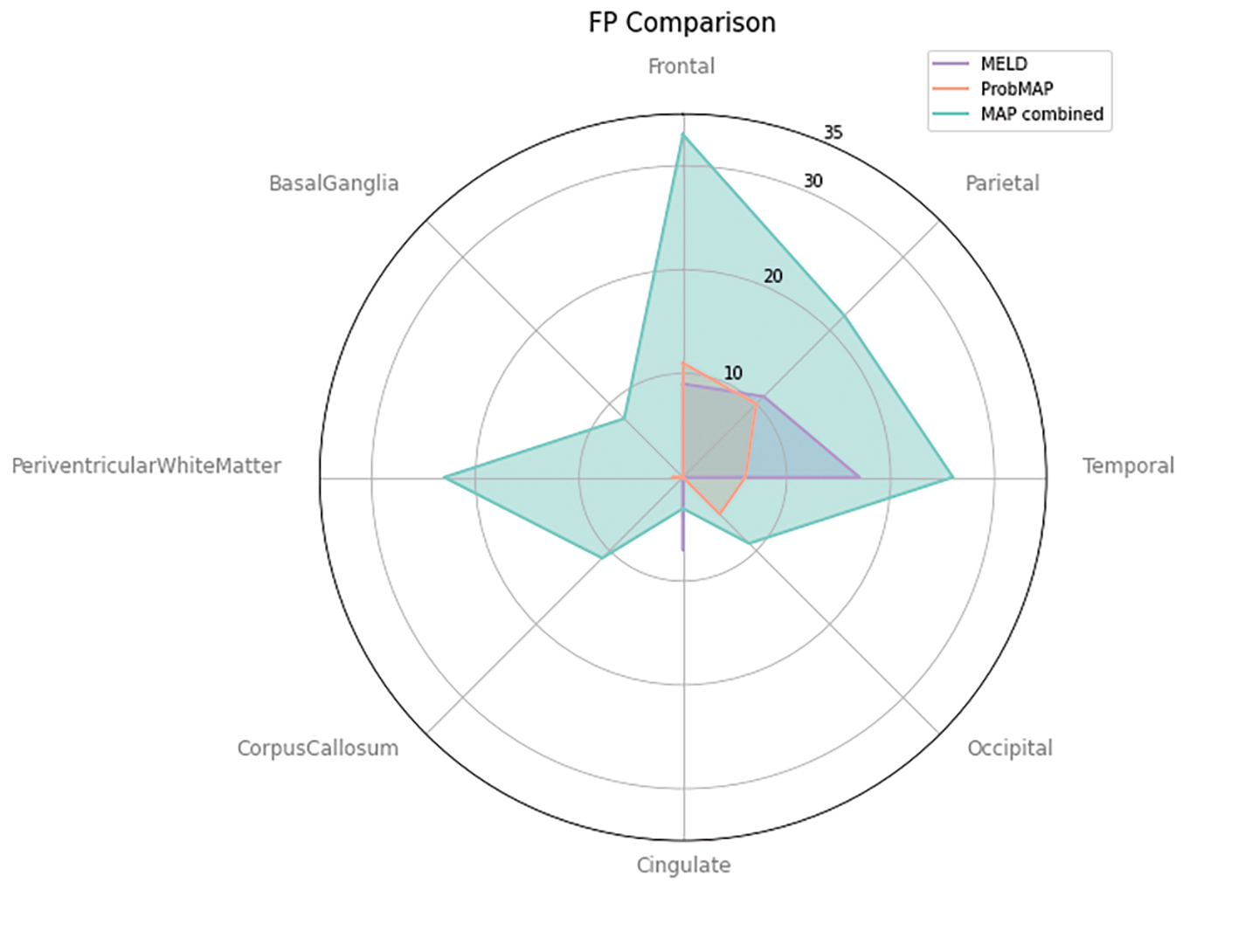

Supplement: Supplementary file 1 — Supplementary file1 (DOCX 185 KB) [file 234_2025_3549_MOESM1_ESM.docx]
